# Increased Insulin Sensitivity and Distorted Mitochondrial Adaptations during Muscle Unloading

**DOI:** 10.3390/ijms131216971

**Published:** 2012-12-11

**Authors:** Zhengtang Qi, Yuan Zhang, Wei Guo, Liu Ji, Shuzhe Ding

**Affiliations:** 1 Key Laboratory of Adolescent Health Assessment and Exercise Intervention, Ministry of Education, East China Normal University, Shanghai 200241, China; E-Mails: qzht79@163.com (Z.Q.); beibei82506@126.com (Y.Z.); lji@tyxx.ecnu.edu.cn (L.J.); 2 School of Physical Education & Health, East China Normal University, Shanghai 200241, China; 3 Hangzhou Institute of Sports Science, Hangzhou 310004, China; E-Mail: wguo1226@yahoo.cn

**Keywords:** mitochondrial, insulin sensitivity, pyruvate dehydrogenase kinase 4, hindlimb unloading, skeletal muscle

## Abstract

We aimed to further investigate mitochondrial adaptations to muscle disuse and the consequent metabolic disorders. Male rats were submitted to hindlimb unloading (HU) for three weeks. Interestingly, HU increased insulin sensitivity index (ISI) and decreased blood level of triglyceride and insulin. In skeletal muscle, HU decreased expression of pyruvate dehydrogenase kinase 4 (PDK4) and its protein level in mitochondria. HU decreased mtDNA content and mitochondrial biogenesis biomarkers. Dynamin-related protein (Drp1) in mitochondria and Mfn2 mRNA level were decreased significantly by HU. Our findings provide more extensive insight into mitochondrial adaptations to muscle disuse, involving the shift of fuel utilization towards glucose, the decreased mitochondrial biogenesis and the distorted mitochondrial dynamics.

## 1. Introduction

Hindlimb unloading (HU) is frequently used to simulate and study neuromuscular perturbations occurring in disuse-induced muscle wasting and atrophy [1–4]. The major modifications concerning HU are muscle atrophy [5,6] and slow-to-fast fiber type transition [7,8]. Consequently, unloading-induced muscle demonstrates an increased utilization on glucose and a corresponding decreased use of lipid [9]. This conversion of fuel source is associated with muscle fiber type switching and further results in activation of glycolysis and inhibition of fatty acid oxidation in unloaded skeletal muscle [10]. Striated muscles differ in mitochondrial content, and muscle fiber type switching involves changes in mitochondrial content. Type I (slow-twitch) fibers are much higher in mitochondrial content and are more dependent on oxidative metabolism than type II (fast-twitch) fibers. The pronounced differences in mitochondrial content between fiber types could be attributed to a combination of differences in myonuclear domain and modest effects on the expression of nuclear and mitochondrial encoded respiratory genes [11]. PGC-1α has been identified as a principal factor regulating muscle fiber type determination [12]. Skeletal muscle-specific PGC-1α knock-out mice exhibit a shift from oxidative type I and IIa toward type IIx and IIb muscle fibers [13]. However, an important study suggests that PGC-1 coactivators (α and β) are necessary for the oxidative and mitochondrial programs of skeletal muscle, but are dispensable for fundamental fiber type determination and insulin sensitivity [14]. Skeletal muscle contractile activity, such as endurance exercise, also leads to a variety of physiological and biochemical adaptations in skeletal muscle, including mitochondrial biogenesis, angiogenesis and fiber type transformation [15]. PGC-1α also plays a functional role in exercise-induced mitochondrial biogenesis and angiogenesis, but not IIb-to-IIa fiber-type transformation in skeletal muscle, and the improvement of mitochondrial morphology and antioxidant defense in response to endurance exercise may occur independently of PGC-1α function [16]. In the adaptation to chronic exercise, PGC-1α reduces maximal force, increases resistance to fatigue and drives fiber type switching. partly through remodeling of calcium transients, in addition to promoting slow-type myofibrillar protein expression and adequate energy supply [17]. Relatively less attention has been paid to studying the responses of PGC-1α and mitochondrial consequence in the unloaded skeletal muscle.

Mitochondrial dynamics responds to changes in energy demand and cell division by the equilibrium between fusion and fission. Several important regulatory proteins in mammal have been identified in the processes: dynamin-related protein-1 (Drp1) and fission-1 (hFis1), essential for fission; mitofusins (Mfn1, Mfn2); and optical atrophy-1 (OPA1), essential for fusion. All of them, except hFis1, are GTPase as a dynamin mediating dynamics of mitochondrial inner/outer membrane by hydrolyzing GTP. Mfn2 expression is driven by a PGC-1α coactivation with estrogen-related receptor α (ERRα), which is also essential for the expression of genes involved in mitochondrial biogenesis, antioxidant protection and oxidative phosphorylation [18,19]. The PGC-1α/ERRα induction of Mfn2 suggests that PGC-1α not only mediates the increased expression of oxidative phosphorylation genes, but also mediates alterations in mitochondrial fusion in response to exercise [20]. A regulatory pathway involving PGC-1α, ERRα and Mfn2 participates in the pathophysiology of insulin-resistant conditions and type 2 diabetes [21]. Interestingly, ERRα is also a specific partner of PGC-1α for the activation of PDK4 expression in muscle [22], suggesting a mechanism, whereby PGC-1α exerted reciprocal inhibitory influences on glucose catabolism, while increasing alternate mitochondrial oxidative pathways [23]. Therefore, mitochondrial biogenesis and fusion, fuel utilization and muscle fiber-type switching are interconnected and required as a whole to respond to changes in energy demands during muscle exercise or disuse. This study aimed to investigate the mitochondrial consequence and the expression of nuclear-encoded genes involved in mitochondrial biogenesis, mitochondrial dynamics and fuel utilization in the unloaded skeletal muscle. Further investigation of these adaptive mechanisms may extend our understandings of skeletal muscle plasticity and provide novel insights into both spaceflight- and muscle disuse-related atrophy.

## 2. Results

### 2.1. Gastrocnemius Relative Mass and Blood Metabolic Parameters

**Figure 1:**
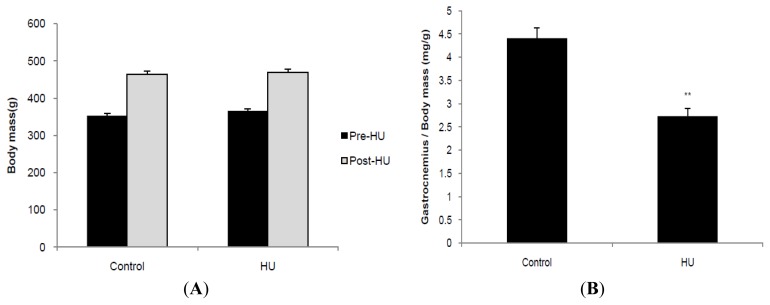

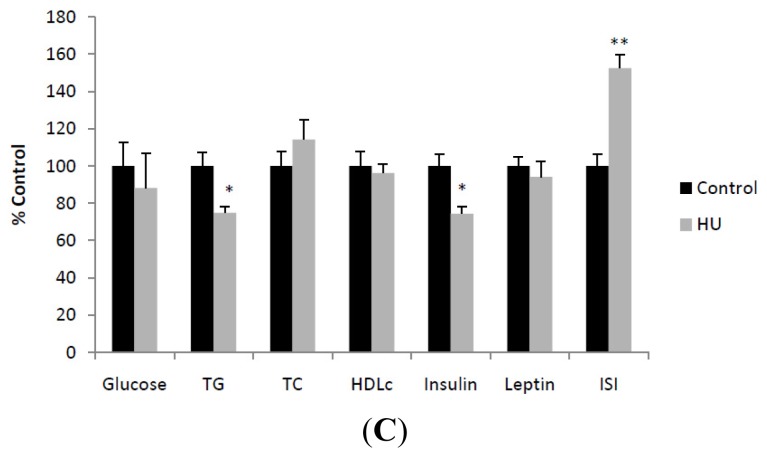
Body mass (**A**), muscle relative weight (**B**) and blood parameters (**C**) in rats subjected to 21-day hindlimb unloading(HU). TG, triglyceride; TC, total cholesterol; HDLc, high-density lipoprotein cholesterol; ISI, insulin sensitivity index (= 1/(FPG × FINS)). Values are means ± SE. *n* = 8~10 for each group. *p < 0.05, **p < 0.01 *vs.* control rats.

HU had no significant effect on body mass, but significantly decreased the wet weight of gastrocnemius relative to body mass (Figure 1A,B). HU had no significant effect on blood levels of glucose, TC, HDLc and leptin, but significantly decreased blood levels of TG and insulin (Figure 1C). Thus, insulin sensitivity index (ISI) was calculated by a formula: ISI = 1/(blood glucose × insulin). HU increased ISI significantly (Figure 1C).

### 2.2. Mitochondrial Content and Oxidative Damage

**Figure 2:**
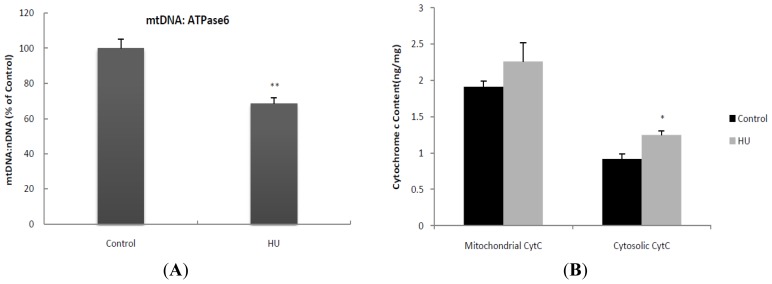

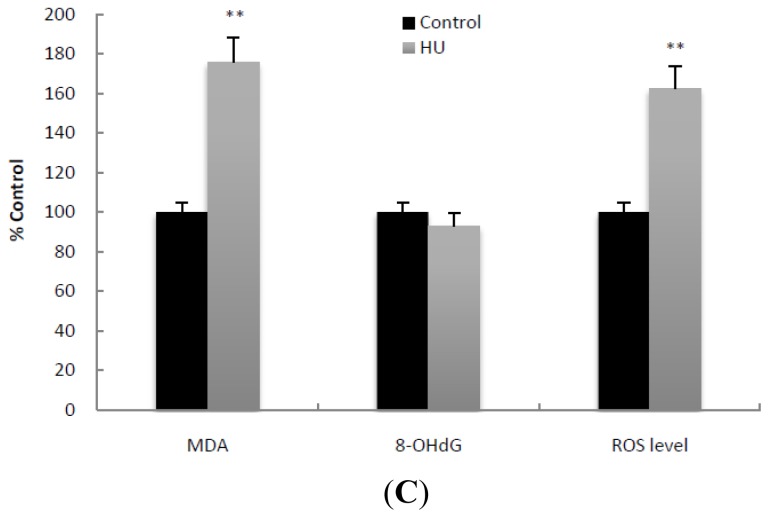
Mitochondrial DNA content (**A**), Cytochrome c content (**B**) and oxidative damage (**C**) in skeletal muscle subjected to 21-day hindlimb unloading (HU). Real-time PCR was used to determine mtDNA content in gastrocnemius, relative to the endogenous control β-actin. ATPase6 was used as mtDNA marker, and β-actin was used as a nuclear DNA marker. Values are means ± SE. *n* = 6 for each group. * *p* < 0.05, ** *p* < 0.01 *vs.* control rats.

mtDNA was quantified by PCR, Cytochrome c content, and MDA, 8-OHdG and ROS level were measured in the isolated mitochondria. mtDNA content was significantly decreased in the HU case compared to the control, whereas Cytochrome c in the mitochondrial fractions was not increased by HU (Figure 2A,B). Remarkably, we found here that HU significantly increased Cytochrome c content in the cytosol (Figure 2B). A pivotal role has been ascribed to oxidative stress in determining the imbalance between protein synthesis and degradation leading to muscle atrophy in many pathological conditions and in disuse. However, a large variability in the disuse-induced alteration of redox homeostasis through muscles, models and species emerges from the literature [24]. In the current study, HU increased MDA and ROS level in the mitochondrial fractions isolated from muscle, whereas 8-OHdG level was unaltered (Figure 2C).

### 2.3. Expression of Genes Involved in Fuel Utilization, Mitochondrial Biogenesis and Dynamics

**Figure 3:**
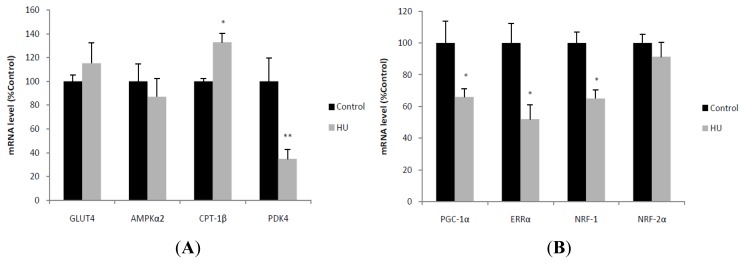

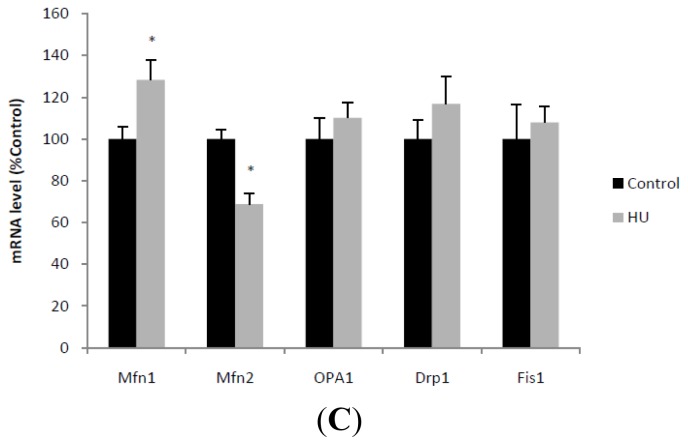
mRNA quantification of proteins and regulators controlling fuel utilization (**A**), mitochondrial biogenesis (**B**) and dynamics (**C**) in skeletal muscle subjected to 21-day hindlimb unloading (HU). Real-time PCR was used to determine expression of genes in gastrocnemius from HU and control rats, relative to the endogenous control β-actin. Values are means ± SE. *n* = 6 for each group. * *p* < 0.05, ** *p* < 0.01 *vs.* control rats.

PDK4 transcript level was significantly decreased by HU, whereas CPT-1beta mRNA was significantly increased. The mRNA levels of GLUT4 and AMPK α2 were not changed by HU (Figure 3A). As to mitochondrial biogenesis and regulation of oxidative phosphorylation genes, the mRNA levels of PGC-1α, ERRα and NRF-1 were significantly decreased by HU (Figure 3B). As to mitochondrial fusion and fission, Mfn2 transcript level was significantly decreased by HU, whereas Mfn1 mRNA was significantly increased. The mRNA levels of OPA1, Drp1 and Fis1 were not changed by HU (Figure 3C).

### 2.4. Mitochondrial Protein Level of PDK4, CPT-1beta, Mfn2 and Drp1

**Figure 4:**
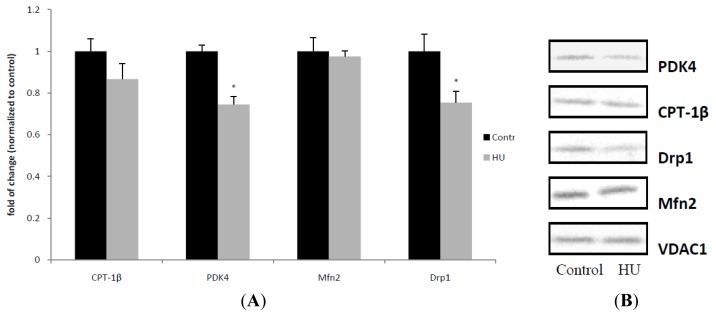
Western blot analysis and densitometric ratios of PDK4, CPT-1β, Drp1 and Mfn2 in mitochondrial fractions. Western blot analysis was used to determine the level of proteins in mitochondrial fractions extracted from gastrocnemius, relative to the endogenous VDAC1 (mitochondrial). Values are means ± SE (**A**). Western blots (**B**) are representative from one rat from each group. *n* = 4 for each group. * *p* < 0.05 *vs.* control rats.

To further identify the level of mitochondrial proteins involved in fuel utilization and mitochondrial dynamics, mitochondrial fractions were analyzed by Western blot. HU significantly decreased the level of PDK4 and Drp1, and the protein levels of CPT-1beta and Mfn2 were not changed by HU (Figure 4A,B).

## 3. Materials and Methods

### 3.1. Animals and Hindlimb Unloading

Male Sprague Dawley rats weighing 180–210 g were obtained from Shanghai SLAC Experimental Animal Center (Shanghai, China). After a five-week accommodation in our animal house, all rats were randomly assigned to one of two groups: (1) Control and (2) Hindlimb unloading (HU). All of the animals received a special food for rodents (No. M01, Shanghai SLAC, China) and purified water for drinking. Control rats were housed three-to-four per cage in a temperature-controlled environment maintained at 22–25 °C with a 12:12 h light-dark cycle. The animals assigned to the HU group were achieved by use of a tail harness to suspend the hindlimbs 1–2 cm above the floor, as described previously [25]. The animals were prevented from having the hindlimb touch the grid floor or other supportive surfaces for three weeks. During this period, the rats were able to move about in a circular area, eat food and drink water freely by using their forelimbs. All experimental procedures and animal care were approved by the Experimental Animal Care and Use Committee at East China Normal University (License number for the use of laboratory animals: SYXK (Shanghai) 2004-0001) and followed the Guiding Principles for the Care and Use of Laboratory Animals established by the People’s Republic of China Ministry of Health (25 January 1998).

### 3.2. Tissue Dissection and Blood Sampling

All animals were fasted for 6 h and sacrificed rapidly by cervical dislocation. Blood was collected via cardiac puncture. Surgical removal of gastrocnemius was accomplished within 10 min. Blood was transferred to a microcentrifuge tube, where it was allowed to clot (25–30 min) before being centrifuged (3000 rpm for 10 min at 4 °C) and the serum collected. Serum insulin and leptin were assayed using ELISA specific kits (San Diego, CA, USA) and measured on a TECAN Micropalte Reader (Infinite 200, Mannedorf, Switzerland) at 450 nm according to the manufacturer’s protocol. Blood glucose, triglyceride, total cholesterol (TC) and high density lipoprotein cholesterin (HDLc) were assayed using Nanjing Jiancheng kits (Jianchen Biochemical Inc, Nanjing, China) and measured on TECAN Micropalte Reader, according to the instructions included. Insulin sensitivity index (ISI) was calculated by a formula: ISI = 1/(blood glucose × insulin).

### 3.3. Subcellular Fractionation and ELISA for Cytochrome C and 8-OHdG

Subcellular fraction was prepared using differential centrifugation as previously described [26]. The muscle sample was homogenized in ice-cold isolation buffer (210 mM mannitol, 70 mM sucrose, 5 mM Tris-HCl, 1 mM EDTA, 0.5 mM DTT, 0.1 mM PMSF; pH 7.4) at a weight/volume ratio of 1:10. The homogenate was centrifuged at 1300*g* for 10 min at 4 °C. The supernatant (S1) was carefully removed from the pellet (P1). The S1 was further centrifuged at 17,000*g* for 15 min at 4 °C. The supernatant resulting from this procedure (S2) was carefully removed from the pellet (P2) and centrifuged at 17,000*g* for 15 min at 4 °C to remove residual mitochondria. The resulting supernatant (S3) was designated the mitochondrial-free, nuclear-free, cytosolic-enriched protein fraction for analysis. The P2 containing mitochondria was washed (17,000*g* for 15 min at 4 °C) twice in 200 μL mitochondrial isolation buffer (210 mM mannitol, 70 mM sucrose, 5 mM Tris-HCl, 1 mM EDTA, 0.5 mM DTT, 0.1 mM PMSF; pH 7.4). The pellet was resuspended in mitochondrial isolation buffer, lysed by three consecutive freeze-thaw cycles and sonicated on ice for 20 s. The suspension was designated the mitochondrial-enriched fraction for analysis. Subcellular protein concentrations were determined by the BCA assay. Cytochrome C(CytC) and 8-OHdG were assayed using ELISA-specific kits (San Diego, CA, USA) and measured on a TECAN Micropalte Reader, according to the manufacturer’s protocol. MDA was assayed in mitochondrial fractions using Nanjing Jiancheng kits (Jianchen Biochemical Inc, Nanjing, China) and measured on the TECAN Micropalte Reader according to the instructions included. Mitochondrial ROS level was detected using H2DCF-DA. Fluorescence was determined with a fluorescence spectrophotometer at 488 nm (excitation) and 525 nm (emission), as described previously [27].

### 3.4. Immunoblotting

For Mfn2, Drp1, PDK4 and CPT-1β, mitochondrial-enriched fractions were isolated as described above, VDAC1 (Voltage-dependent anion channel 1) was used to standardize the amount of protein loaded. The polyclonal antibodies used were obtained from Santa Cruz Biotechnology. The following antibodies were used: Mfn2: sc-100560, Drp1: sc-32898, CPT-1β: sc-20670, PDK4: sc-14492. The protein samples were diluted in buffer to the same concentration (1 g/L). Equal amounts of protein (30 μg/lane) were run on 12% SDS-polyacrylamide (120 V; Bio-Rad, Hercules, CA, USA), and proteins were transferred (1 h, 1.2 mA/cm^2^, Criterion blotter; Bio-Rad, Hercules, CA, USA) to PVDF membranes. Visualization of bands was performed by 3,3'-DAB staining (Shanghai Sangon, Shanghai, China) and scanned densitometrically, and quantification was performed with Gel image-processing system.

### 3.5. Real-time PCR and mtDNA Quantitation

Total RNA was prepared from ~100 mg of frozen muscle tissues using TRIzol (Invitrogen, Chromos, Singapore) and purified according to the instructions included. RNA purity was verified by the OD_260_/OD_280_ on the ultraviolet spectrophotometric module of the TECAN Micropalte Reader (Infinite 200, Mannedorf, Switzerland). Double-stranded cDNA was synthesized from ~1 μg of total RNA using ReverTra Ace^®^ qPCR RT Kit (TOYOBO CO., LTD, Osaka, Japan). Real-time PCR reactions were set up using the SYBR-Green PCR kit (TOYOBO, Osaka, Japan) and were cycled in StepOne™ Real-Time PCR System (Applied Biosystems, Foster City, CA, USA); the mRNA abundance of the targeted genes was normalized to that of β-actin. Mitochondrial DNA content was also determined by qPCR. Briefly, mtDNA and total DNA were extracted and purified from tissues by the method described previously [28]. To quantify the amount of mtDNA present per nuclear genome, we used the following primers: ATPase6. To quantify nuclear DNA, we used a primer set that detects the β-actin. Quantification of relative copy number differences was carried out using analysis of the difference in threshold amplification between mtDNA and nuclear DNA. PCR was performed as stated above. Primer pairs were designed based on GenBank reference sequences and listed in Supporting Material for the article Table S1.

### 3.6. Statistical Analysis

All data were presented as mean ± SE. All differences were analyzed by the student’s *t*-test. For all analyses, *p* value <0.05 was considered statistically significant.

## 4. Discussion

Our findings provide the insight that skeletal muscle adaptations to HU may be associated with the decreased expression of PDK4, PGC-1α, ERRα, NRF-1 and Mfn2, resulting in changes in mtDNA content, mitochondrial network and fuel utilization. Interestingly, HU increases the insulin sensitivity in the whole body.

As a whole, 21 days of HU did not induce a significant metabolic disorder, except a significant loss of gastrocnemius relative to body weight. In agreement with our results, it was reported in 1997 that hindlimb suspension increased basal glucose transport, lactate production and glycogen synthesis. An increase in the sensitivity of these processes to insulin occurred as early as 24 h and persisted for five weeks of the muscle unloading. However, the authors emphasized that the enhanced glucose utilization and improved muscle insulin sensitivity during hindlimb suspension were not related to muscle atrophy, because muscle atrophy was not observed in the early stage of muscle unweighting [29]. An earlier study in 1988 showed that the increased sensitivity to insulin of the unloaded soleus was associated with the degree of muscle atrophy, due to an increased insulin binding capacity relative to muscle mass [30]. In the recent decade, little attention was focused on the relationship between the increased insulin sensitivity and unloaded muscle, likely because these results led us into a paradox that chronic muscle use and disuse could both increase insulin sensitivity. In contrast, ROS was focused intensively for its potential roles in muscle atrophy and insulin sensitivity. We also reported here that HU increased MDA level and ROS production in mitochondria. Although it is feasible that increased ROS production in muscle fibers can promote proteolysis and also depress protein synthesis during periods of skeletal muscle inactivity, and it is established that oxidants can participate in the regulation of protein turnover in cells, there remains a debate as to whether oxidative stress is required for disuse skeletal muscle atrophy. The mere coexistence of muscle atrophy, indirect indexes of increased ROS production and impairment of antioxidant defense systems, in fact, could not unequivocally support a causal role of oxidative stress in disuse-induced muscle loss [24]. Recently, the positive role of ROS in mitochondrial biogenesis and insulin action was also well established. For instance, mice lacking glutathione peroxidase 1 (Gpx1), characterized by increased insulin sensitivity, were protected from high-fat-diet-induced insulin resistance [31]. Vitamin E, C and alpha-lipoic acid supplementation suppressed skeletal muscle mitochondrial biogenesis, regardless of training status [32,33]. Therefore, ROS production and oxidative stress may be a mediator necessary, but not sufficient enough, to determine muscle loss and insulin sensitivity in the hindlimb-unloaded rats.

In unloaded muscle, fuel uptake shifts from FA towards carbohydrate [9,10], and slow-to-fast fiber-type switching [7,34] were reported frequently in the previous studies. Further, the shift of slow muscle toward fast type by unloading was associated with a decrease in mitochondrial biogenesis [35]. With contractile activity, the type I fiber from the HU animal showed a greater utilization of glycogen and accumulation of lactate compared with the control type I fiber [36]. However, the associated mechanisms remain poorly defined. We first reported here that HU decreased PDK4 expression and its protein level in mitochondria, whereas CPT-1beta expression was increased by HU. Expression of AMPKα2 and GLUT4 was not changed by HU. Presumably, PDK4 was selected for skeletal muscle adaptations to HU. PDK4 is not only the negative regulator of glucose oxidation, but also a direct target of PPARdelta/beta, FoxO1 and ERRα [23]. CD36-mediated FA uptake can up-regulate protein levels and activity of FoxO1, as well as its target PDK4. FoxO1 in turn can regulate CD36, lipoprotein lipase and PDK4, reinforcing the action of PPARdelta/beta to increase muscle reliance on FA [37]. The forkhead transcription factor (FoxO1) binds the PDK4 gene and contributes to the induction of PDK4 by ERRs and PGC-1α [38]. In this study, due to the negative regulation of PDK4 on glucose oxidation, decreased PDK4 may underlie the shift of fuel utilization in the unloaded muscle. In addition, the decreased expression of ERRα and PGC-1α suggests that the transcriptional pathway including PGC-1α, ERRα and PDK4 is downregulated by HU. As reported previously, PDK4 activity, protein and mRNA were lower in glycolytic *vs.* oxidative myofibers [39]. PGC-1α overexpression increased the oxidation rate of palmitic acid and mRNA expression of genes regulating lipid metabolism, mitochondrial biogenesis and function in myotubes. Basal and insulin-stimulated deoxyglucose uptake were decreased, possibly due to upregulation of PDK4 mRNA. Expression of fast fiber-type gene marker (MHCIIa) was decreased [40]. Herein, the decreased PDK4 may be an essential biomarker of the slow-to-fast fiber-type transition during HU, in combination with the decreased expression of PGC-1α and the potential shift of fuel utilization in skeletal muscle. Additionally, hindlimb unloading induced severe muscle atrophy in soleus muscle, but only slight atrophy in EDL muscle. The concentration of insulin receptor substrate-1 (IRS-1) and phosphorylation of IRS-1 at Ser636-639 and Ser789 were also reduced in soleus muscle due to unloading [41]. Unloading stress also resulted in skeletal muscle atrophy through the induction and activation of the ubiquitin ligase Cbl-b. Cbl-b-dependent destruction of IRS-1 is a critical dual mediator of both increased protein degradation and reduced protein synthesis observed in unloading-induced muscle atrophy [42]. These findings might also be responsible for the abnormal insulin actions in unloaded muscle. However, the increase in blood glucose uptake independently of insulin is probable, because it often occurs during exercise (muscle use).

HU decreased mtDNA content and distorted the expression of mitochondrial dynamics machinery. ELISA assays showed no significant changes in mitochondrial Cytochrome C content, whereas qPCR indicated mtDNA content was reduced by HU. This finding is inconsistent with that previously reported by Pesce *et al.*, who showed the mtDNA content did not change in three-month-old, but decreased significantly in 15-month-old, rats after 14 days of HU [43]. We also observed down- regulation of PGC-1α, ERRα and NRF-1 after HU. In contrast to our findings, Wagatsuma *et al.* reported that PGC-1α was up-regulated concomitant with decreased expression of its DNA binding transcription factors, PPARα and ERRα, after a week of HU. Moreover, there was no alteration in expression of NRF-1, but NRF-2 was up-regulated [44]. Oishi *et al.* also reported that the levels of heat shock proteins Hsp)60 and Hsp72, mitochondrial Cytochrome C oxidase subunit IV (Cox IV) and PGC-1 proteins were decreased after two weeks of HU [45]. Anyway, our findings in the transcriptional control were consistent with the reduction in mtDNA content after HU and further implied a role of PGC-1α signaling in the muscle fiber-type switching.

The overall structure of the mitochondrial population depends on the relative activities of the two sets of proteins mediating fusion and fission. Herein, HU increased Mfn1 expression and decreased Mfn2 expression and the Drp1 protein level. Importantly, the coiled-coil (CC) domain of Drp1 alone targets specifically and exclusively to mitochondria, implicating its involvement in localizing Drp1 to this organelle *in vivo*[46]. Drp1 is recruited to fission spots on the membrane by hFis1 during mitochondrial fission [47]. Expression of the fission machinery is sufficient to cause muscle wasting in adult animals by triggering mitochondrial dysfunction. Conversely, inhibition of the mitochondrial fission inhibits muscle loss during fasting and after FoxO3 overexpression [48]. Thus, we concluded here that Drp1 assembled on mitochondria was decreased by HU, suggesting a putative reduction of mitochondrial fission. Both Mfn1 and Mfn2 are essential for mitochondrial fusion [49], but mRNA expression of them appeared distorted by HU. Compared to Mfn1, Mfn2 is independent of its role as a mitofusin, and it can also trigger mitochondrial energization by regulating OXPHOS expression [50]. Mfn2 reduction decreased the rate of fatty acid synthesis in the liver, and the Mfn2/shRNA mice exhibited hypertriglyceridema, suggesting that Mfn2 played an important role in maintaining glucose and lipid homeostasis [51]. Repression of Mfn2 reduced glucose oxidation, mitochondrial membrane potential, cell respiration, and mitochondrial proton leak and reduced Mfn2 expression may explain some of the metabolic alterations associated with obesity [52]. PGC-1β is a regulator of normal expression of Mfn2 in muscle, whereas PGC-1α participates in the stimulation of Mfn2 expression under a variety of conditions characterized by enhanced energy expenditure [53]. These observations suggest the existence of interconnection responsible for the abnormal control of the expression of genes encoding for modulators of mitochondrial biogenesis, dynamics and metabolism and which may participate in the development of the disease [54]. Therefore, the concerted expression of PGC-1α and Mfn2 may be necessary for mitochondrial adaptations to HU, including mitochondrial biogenesis, dynamics and metabolism. In the current study, Mfn2, concomitant with decreased expression of PGC-1α, ERRα and NRF-1, may play a role as a regulator or effector more than a mitofusin in skeletal muscle adaptations to HU.

## 5. Conclusions

Our findings provide more extensive insight into skeletal muscle adaptations to HU, involving the increased glucose utilization, the decreased mitochondrial biogenesis and the distorted mitochondrial dynamics. The reduced PDK4 during muscle unloading may lead to the shift of fuel utilization and muscle-fiber type. Interestingly, HU increased the capacity of insulin to control blood glucose, whereas mitochondrial biogenesis and dynamics was decreased or distorted in skeletal muscle. However, the ability of muscle unloading to improve insulin resistance cannot be further expected, due to the muscle loss during HU. Further studies are required to investigate whether and how the slow-to-fast fiber-type switching increases glucose utilization during muscle unloading.

## Supplementary Information
