# Supplementary material for: Increased Insulin Sensitivity and Distorted Mitochondrial Adaptations during Muscle Unloading
Source: Int J Mol Sci. 2012 Dec 11;13(12):16971–85. doi: 10.3390/ijms131216971 (PMC3546734; doi:10.3390/ijms131216971)
Supplement: Supplementary file 1 [file ijms-13-16971-s001.pdf]

Supplementary Information

Table S1. Gene primers sequences for real-time PCR (5' to 3').

Gene	Forward Primer	Reverse Primer	UniSTS#
GLUT4	GACATTTGGCGGAGCCTAAC	TAACTCCAGCAGGGTGACACAG	239613
AMPKα2	GAGAGAGGACTCGGCATCAATAA	AAGGAAACAGGCAGATACCAACA	231697
PDK4	CCAGCACATCCTCATATTCAGTG	GAGCTTTAATTCTGGCGACGTTA	231631
CPT-1β	ATCTCGGTTCCAGTTCTACTTCC	ACGACAGTCTCACTTAGAGGCAC	261874
PGC-1α	TGCAGGCCTAACTCCTCCCAC	AATAGGCCATCCATGGCTAGTCC	479198
ERRα	AGTACAGCTGTCCGGCCTCCAAC	GGCATGGCATACAGCTTCTCAGG	277893
NRF-1	TGAGGGCATTGGATTTGGATTA	GGGAGAGGCAAGAGAATCACTC	218742
NRF-2α	AGCGCATCTCGTTGAAGAAG	CCGAAATGTTGAGTGTGGTG	259081
Mfn1	GCTGCATACAGACAGACAGCCT	GGTAATGACCTGTCTCAGGGCT	218000
Mfn2	CACTACCACATCGGACACCCTA	GAACTTGTGTCTTGCATTTGGC	211475
OPA1	TGGCCCATTCATAGAGACAG	CCTTTATGACTGGCGGAGAA	226398
Drp1	GAAGTGGTGCAGTGGAAATGAC	GTTTCTATTGGGAACCACTGCC	214962
hFis1	AAATGATGCTACGCAGGCTT	CCTGGACCATGACCAAGTTT	232123
ATPase6	CAAACAAATAATGCTAATCCACACACC	GCTGTAAGCCGGACTGCTAATG	
β-actin	CCTCTATGCCAACACAGTGC	GTACTCCTGCTTGCTGATCC	

GLUT4, glucose transporter 4; AMPKα2, AMP-activated protein kinase subunit α2; PDK4, pyruvate dehydrogenase kinase 4; CPT-1β, carnitine palmitoyltransferase 1(muscle); PGC-1α, proliferator-activated receptor-γ coactivator-1α; ERRα, estrogen-related receptor α; NRF-1, nuclear respiratory factor-1; NRF-2α, nuclear respiratory factor-2α; Mfn1 and Mfn2, mitofusin 1 and 2; OPA1, optic atrophy 1; Drp1, dynamin-related protein 1; hFis1, mitochondrial fission protein. ATPase6, mitochondrial adenosine triphosphatase 6.

© 2012 by the authors; licensee MDPI, Basel, Switzerland. This article is an open access article distributed under the terms and conditions of the Creative Commons Attribution license (http://creativecommons.org/licenses/by/3.0/).
